# Organ and tumour dosimetry of ^177^Lu-rhPSMA-10.1, a novel PSMA-targeted therapy: results from a Phase I trial

**DOI:** 10.1007/s00259-025-07313-z

**Published:** 2025-05-06

**Authors:** James Nagarajah, Hyun Kim, Luke Nordquist, Vikas Prasad, Nathaniel Scott, Daniel Stevens, Benjamin Fongenie, Joseph Osborne

**Affiliations:** 1 Radboud University Nijmegen Medical Centre, Nijmegen, The Netherlands; 2 Roentgeninstitut Düsseldorf, Düsseldorf, Germany; 3 Washington University School of Medicine, St Louis, MO US; 4 XCancer Corporation, Omaha, NE USA; 5 Blue Earth Therapeutics Ltd, Oxford, UK; 6 Molecular Imaging and Therapeutics, Department of Radiology, Weill Cornell Medicine, New York, NY US; 7 Department of Radiology and Nuclear Medicine, Radboud University Nijmegen Medical Centre, P.O. Box 9101, Nijmegen, 6500 HB The Netherlands

**Keywords:** Dosimetry, Pharmacokinetics, Prostate cancer, Prostate-specific membrane antigen, Radioligand therapy

## Abstract

**Purpose:**

To evaluate tumour and normal organ dosimetry of PSMA-targeted RLT ^177^Lu-rhPSMA-10.1.

**Methods:**

PSMA-positive mCRPC patients experiencing disease progression following standard-of-care treatment were enrolled and underwent ≤ 3 cycles of 5.55 or 7.40 GBq ^177^Lu-rhPSMA-10.1 at 6-week intervals. Multi-bed SPECT/CT was conducted 3-, 24-, 48-, and 168-hours post-administration to calculate tumour and organ absorbed doses. Two methods (activity- and anatomy-based) were used for selecting and delineating tumours for dosimetry. Venous blood was collected for radioactivity measurement 30 min before ^177^Lu-rhPSMA-10.1 administration, and 0.5-, 1.5-, 4-, 24-, and 48-hours post-administration.

**Results:**

Thirteen patients were enrolled; three received 5.55 GBq/cycle and 10 received 7.40 GBq/cycle. Mean absorbed doses were 0.266, 0.130 and 8.87 Gy/GBq in kidneys, salivary glands, and tumours (activity-method), respectively, giving mean tumour-to-kidney and tumour-to-salivary ratios of 32.1 and 73.2, respectively. Tumour dose estimates were consistently higher with the activity-method vs. anatomy-method. Tumour absorbed doses decreased each cycle; Cycle 2 and 3 doses were ~ 37% and ~ 56% lower than Cycle 1 estimates, respectively. ^177^Lu-rhPSMA-10.1 was rapidly cleared from the blood (effective half-life, 2.2 h). Imaging data showed mean effective half-lives to be 91.4, 33.7 and 45.4 h in tumours, kidneys, and salivary glands, respectively.

**Conclusion:**

^177^Lu-rhPSMA-10.1 delivers high radiation doses to tumours vs. normal organs, facilitated by its favourable pharmacokinetics. Cumulative doses to normal organs were well within established tolerable limits, suggesting higher cumulative radioactivity could be administered in clinical trials. The observation of decreasing tumour absorbed dose with subsequent cycles also supports the exploration of front-loading radioactivity in Phase II.

**Supplementary Information:**

The online version contains supplementary material available at 10.1007/s00259-025-07313-z.

## Introduction

Prostate-specific membrane antigen (PSMA) is a transmembrane glycoprotein that is overexpressed in prostate cancer cells, particularly in patients with higher grade or metastatic disease [1].

PSMA ligands conjugated with beta-emitters such as 177-lutetium (^177^Lu) have been developed to deliver radiation to PSMA-expressing cells and their surrounding microenvironment. ^177^Lu-PSMA-617 (Pluvicto^®^, Novartis) is the first of these PSMA-targeted radiopharmaceuticals to be approved by the United States Food and Drug Administration for radioligand therapy (RLT) in patients with metastatic castration-resistant prostate cancer (mCRPC) who show disease progression after treatment with anti-androgens and taxane-based chemotherapy [2]. ^177^Lu-PSMA-617 has been shown to extend progression-free survival in men with mCRPC pre- and post-taxane chemotherapy, and to extend overall survival in post-taxane mCRPC patients [3, 4].

A key consideration in PSMA-targeted RLT is that delivering radiation to tumours also means radiation delivery to normal tissue is unavoidable, whether via non-specific means (e.g., circulatory exposure) or through on-target expression– for instance PSMA is expressed to some degree in the healthy kidneys and salivary glands [5, 6]. A further notable consideration is that the known inter- and intra-patient heterogeneity of PSMA expression within tumours [7, 8, 9] can directly influence the delivered radiation dose. Emerging data with ^177^Lu-PSMA-617 suggest that providing a greater radiation dose to the tumour may improve outcomes for patients, and this is further supported by data with other RLT agents such as ^177^Lu-dotatate [10, 11, 12, 13]. However, there remains clinical uncertainty regarding the safe limits of dose to the kidneys which are a key at-risk organ with PSMA-targeted RLT, with decline in renal function and histopathologically-confirmed nephropathy having been documented in patients undergoing ^177^Lu-labelled PSMA RLT [14, 15, 16]. While the risk of delayed radiation nephropathy from RLT is unlikely to impact clinical decision-making for patients with very end stage mCRPC and limited life expectancy, the potential also exists for use of ^177^Lu-labelled PSMA RLT earlier in prostate cancer care (see ongoing trials such as NCT04720157 and NCT06066437). In the future, safe dose limits may depend on the clinical treatment setting, and clinical outcomes may be gated by the tumour-to-kidney absorbed dose ratio (i.e., the therapeutic index). An optimised RLT agent with an improved therapeutic index will allow higher tumour doses to be achieved whilst dosing up to the established safe kidney limits, as well as managing bone marrow exposure and any resulting haematological toxicity. Generating improved RLT agents requires a careful balance of engineering high uptake and long retention in tumours whilst encouraging rapid clearance from plasma and normal tissues. This ideal profile is then paired with a radionuclide that has the right physical half-life to maximise that molecule’s particular profile. Wherever the biological half-life in tumours exceeds the biological half-life in at-risk organs, a longer physical half-life will give an improved therapeutic index, for cumulative radiation dose delivery, compared with a shorter physical half-life [17].

A novel radiohybrid (rh) technology platform has enabled engineering of PSMA-targeted ligands (rhPSMA) that can be labelled with fluorine-18 for diagnostic imaging, or with alpha- or beta-emitting radiometals for RLT [18]. Preclinical assessments with the lead therapeutic candidate, ^177^Lu-rhPSMA-10.1, show it has high PSMA-binding affinity and PSMA-mediated internalisation in LNCaP cells [19]. Moreover, it has finely tuned human serum albumin binding that aims to strike a balance between rapid clearance of the non-PSMA bound fraction of the drug while preserving high tumour accumulation [19].

Comprehensive preclinical evaluations in prostate cancer human xenograft mouse models show ^177^Lu-rhPSMA-10.1 to have a favourable tumour-to-kidney uptake ratio and significant antitumour effects [20, 21]. Furthermore, data from the first patients to receive ^177^Lu-rhPSMA-10.1 under a compassionate use program in Germany show that, within the same patient, ^177^Lu-rhPSMA-10.1 can deliver up to 8-fold greater radiation doses to the tumour than ^177^Lu-PSMA-I&T, with ^177^Lu-rhPSMA-10.1 shown to have a more favourable tumour-to-kidney therapeutic index [22, 23]. Moreover, after treatment with up to six cycles of ^177^Lu-rhPSMA-10.1, patients showed encouraging progression-free survival [24].

Following these initial reports of promising dosimetry and efficacy in patients with mCRPC, formal clinical studies are underway to evaluate the performance of ^177^Lu-rhPSMA-10.1 in larger cohorts. The Phase I/II BET-PSMA-121 study aims to evaluate the safety and antitumour efficacy of ^177^Lu-rhPSMA-10.1 in patients with mCRPC. The Phase I component included comprehensive radiation dosimetry assessments, and here we report absorbed radiation doses in organs and tumours as well as key pharmacokinetic data.

## Methods

### Patients

BET-PSMA-121 (NCT05413850) is an interventional, non-randomised, open-label, integrated Phase I/II study to assess the safety, radiation dosing regimen and antitumour activity of ^177^Lu-rhPSMA-10.1. The current report focuses on radiation dosimetry assessments, specifically, determining the pharmacokinetics, organ-specific and tumour radiation dosimetry of ^177^Lu-rhPSMA-10.1. All other data from the study are reported separately.

The study was conducted in accordance with the Declaration of Helsinki and the International Council on Harmonisation Guidelines for Good Clinical Practice. The protocol was approved by each investigating site’s Independent Ethics Committee prior to initiation and all patients provided written informed consent.

Men > 18 years-old with mCRPC who were experiencing disease progression during or after treatment with at least one novel androgen-axis drug and at least one course (but no more than two courses) of taxane-based chemotherapy were enrolled. Patients were required to have PSMA-positive disease which was established via PSMA PET/CT imaging. PSMA-positive disease was defined as at least one PSMA-positive lesion (bone, lymph node or viscera) of any size with higher uptake than normal liver using visual assessment. At least one PSMA-positive lesion was required to be visible on CT or magnetic resonance imaging and to be greater than 1.5 cm in the short axis. Patients were also required to have no significant PSMA-negative disease detected on baseline cross-sectional imaging (contrast-enhanced CT or magnetic resonance imaging).

**Fig. 1:**
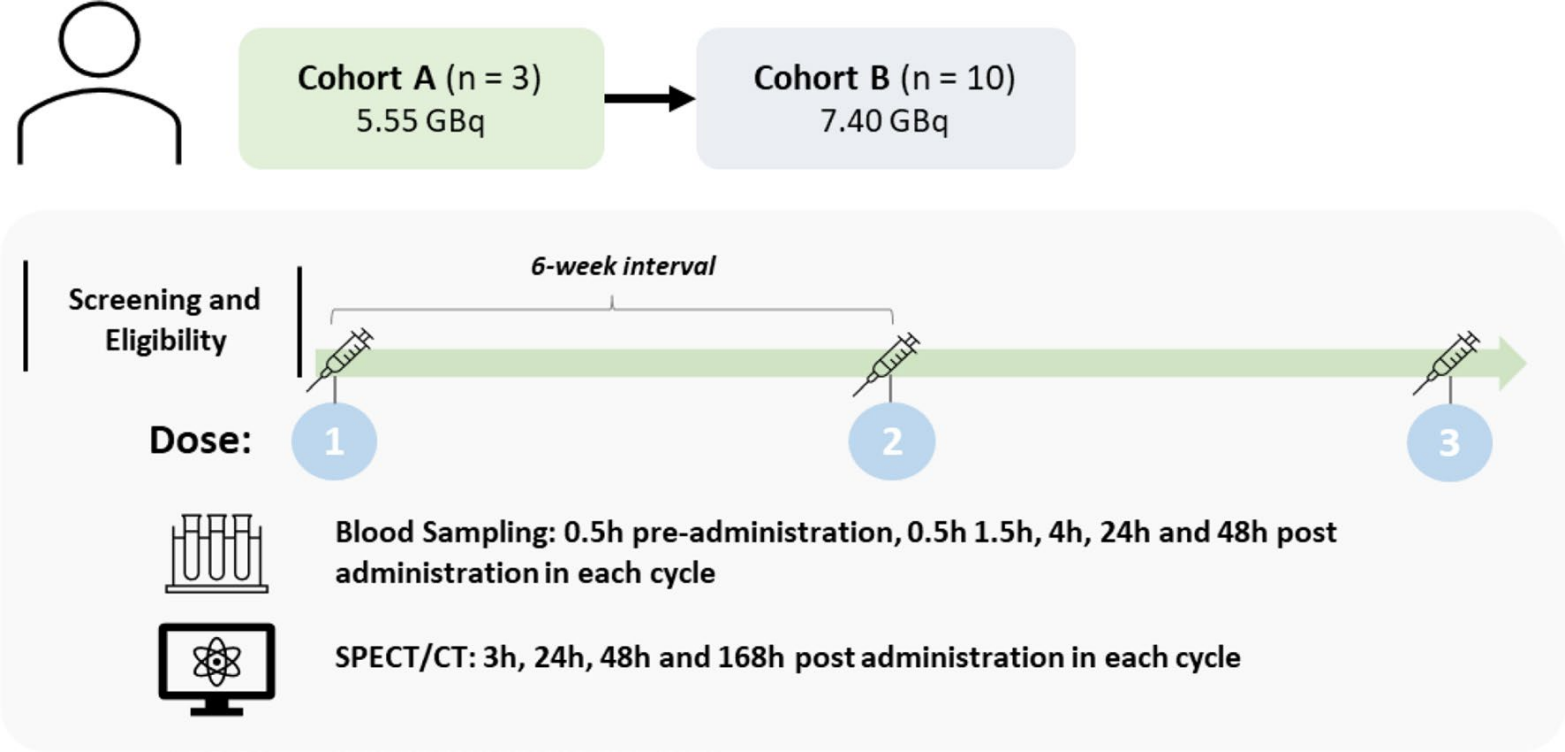
Study Design and Dosimetry Assessments. CT, computed tomography; MIP, maximum intensity projection; SPECT, single-photon emission computed tomography

Figure 1 provides a schematic representation of the study. Enrolled patients were sequentially entered into one of the two cohorts. Cohort A received 5.55 GBq (150 mCi) of ^177^Lu-rhPSMA-10.1 and Cohort B received 7.40 GBq (200 mCi) of ^177^Lu-rhPSMA-10.1. Subsequent doses were delivered at 6-week intervals subject to individual tolerability and safety assessments. Each patient received between one and three cycles, with a maximum of three cycles of treatment being permitted in this initial part of the study. All administrations of ^177^Lu-rhPSMA-10.1 were performed using intravenous injection under the supervision of the treating physician.

### Assessment of pharmacokinetics and dosimetry

To assess the biodistribution and kinetics of radioactivity in individual tissues, whole blood and imaging-based radiation dosimetry were performed. Venous blood samples for radioactivity measurement were collected within the 30 min prior to ^177^Lu-rhPSMA-10.1 administration, and at approximately 30 min, 1.5-, 4-, 24- and 48-hours post-administration in all treatment cycles. Samples were collected from the contralateral arm to that of the ^177^Lu-rhPSMA-10.1 injection. Whole-blood concentrations were measured using a calibrated gamma counter to estimate both red marrow absorbed doses and blood clearance over time. All measurements were decay-corrected to the time of sample collection.

Multi-bed single-photon emission computed tomography (SPECT)/CT was conducted at 3-, 24-, 48- and 168-hours post-^177^Lu-rhPSMA-10.1 administration in each treatment cycle for the estimation of organ and tumour absorbed doses in each patient. Supplemental Table S1 provides details of the image acquisition parameters. Prior to commencement of the study, each investigating site was required to complete a calibration of their dose calibrator and SPECT/CT system to ensure harmonised quantification of the imaging data. Quantitative reconstructions and all dosimetry analyses were carried out centrally by an independent dosimetry vendor (Radiopharmaceutical Imaging and Dosimetry LLC, Baltimore, MD).

The specific absorbed doses (Gy/GBq) in normal organs and tumours were determined using the Medical Internal Radiation Dose (MIRD) Committee S-value methodology [25]. Individual patient organ masses, determined via CT delineation, were scaled to reflect deviations from reference phantom geometry. Cumulative organ absorbed doses (Gy) were calculated across all treatment cycles. Further details on dosimetry methodology are provided in the supplemental materials and have been reported in accordance with EANM guidance [26, 27].

Two approaches were used for tumour selection and delineation for the first treatment cycle only, with the aim of assessing the impact of method on tumour absorbed dose estimates. Approach one was an activity-based method equivalent to that used in most studies that have evaluated tumour dosimetry with ^177^Lu-labelled PSMA RLT [28]. For this method, up to three tumours were selected according to their relative visual uptake on the 168-hour SPECT/CT. Lesion contouring for volume/mass was performed based on a 41% SUV_max_ threshold on the corresponding PSMA PET/CT. These contours were manually transferred onto the SPECT images, making minor adjustments where necessary to the contour margins accounting for any activity spill out on the SPECT. This is illustrated in Supplemental Figure S1. The second method was anatomy-based, with up to five tumours selected in a blinded fashion on CT according to RECIST v1.1 criteria [29]. Lesion volume and mass were determined via manual contouring on the CT and then directly transferred to the SPECT images. Where a common lesion was selected across both methods, the difference in tumour volume resulting from the two contouring methods was calculated.

### Statistical analysis

All continuous data are expressed as the mean (standard deviation [SD]) unless otherwise stated. All blood- and image-based pharmacokinetic analyses were conducted in Python and Excel. Effective half-lives for organs and tumours were calculated using a mono-exponential fit to the washout phase of the time–activity data.

## Results

### Patients

In total, 13 patients were enrolled across four sites in the United States and The Netherlands. Three patients were enrolled into Dosing Cohort A and ten into Dosing Cohort B.

Patients had a mean (range) age of 70 (61–80) years and mean (SD) baseline PSA of 721 (1243) ng/mL (Table 1). In total, two patients received only a single cycle of ^177^Lu-rhPSMA-10.1 therapy, one patient had two cycles, and ten patients received three cycles. The three patients in Cohort A underwent a total of eight cycles of ^177^Lu-rhPSMA-10.1 at 5.55 GBq per cycle and the ten patients in Cohort B underwent a total of 26 cycles of ^177^Lu-rhPSMA-10.1 at 7.40 GBq per cycle.

**Table 1:** Patients’ baseline characteristics

Characteristic	
**Age, years**	
Mean SD Range	70 6.6 61–80
PSA at baseline, ng/mL	
Mean SD	721 1243
**Primary ISUP Grade Group**,** n (%)**	
*3* *4* *5* *Unknown*	3 (23) 6 (46) 2 (15) 2 (15)
**Prior treatment for mCRPC**	
*Taxane (one regimen)* *Taxane (two regimens)* *ARPi/NAAD*	13 (100) 7 (54) 13 (100)
**Administered activity of**^**177**^**Lu-rhPSMA-10.1**,** GBq**	
*Mean* *SD* *Range*	7.1 0.9 5.3–8.0

ARPi, androgen receptor pathway inhibitor; ISUP, International Society of Urological Pathology; mCRPC, metastatic castration-resistant prostate cancer; NAAD, novel androgen axis drug; PSA, prostate-specific antigen; SD, standard deviation

### Dosimetry in normal organs

**Fig. 2:**
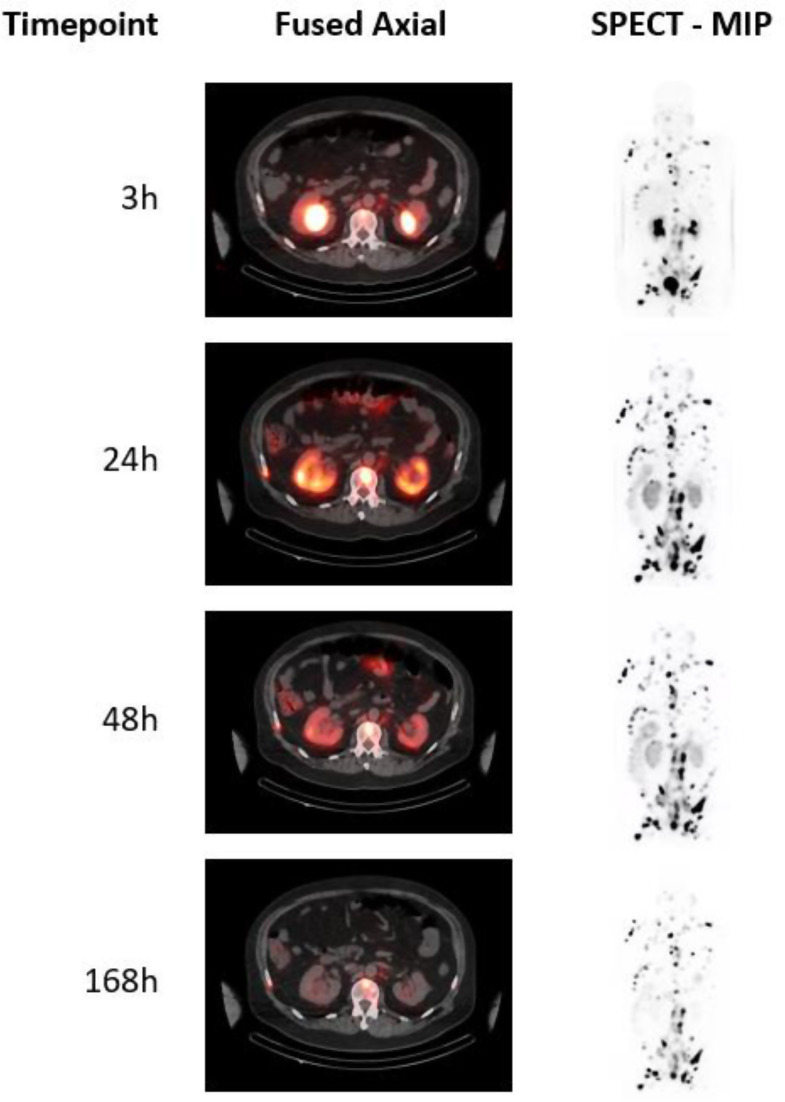
Representative SPECT/CT images show distribution of ^177^Lu-rhPSMA-10.1 at each timepoint after administration (Cycle 1). SPECT images are shown as MIPs. MIP, maximum intensity projection; SPECT, single-photon emission computed tomography

Representative SPECT/CT images from one of the patients in the study are shown in Fig. 2.

Table 2 presents the absorbed radiation dose estimates for the evaluated normal organs for both the first cycle and for all cycles. Of those considered to be key at-risk organs, the mean (SD) absorbed dose of ^177^Lu-rhPSMA-10.1 during Cycle 1 was 0.266 (0.075) Gy/GBq in the kidneys, 0.130 (0.068) Gy/GBq in the salivary glands, and 0.011 (0.010) Gy/GBq (image-based dosimetry) or 0.031 (0.010) Gy/GBq (blood-based methods) in bone marrow. When evaluating the absorbed doses across all cycles, the estimates in these normal organs remained similar (Table 2).

**Table 2:** Absorbed doses per unit activity for normal organs

Organ	Mean (SD) Absorbed Dose, Gy/GBq	
	Cycle 1	All Cycles
Lacrimal glands	0.568 (0.348)	0.611 (0.329)
Kidneys^a^	0.266 (0.075)	0.297 (0.098)
Salivary glands	0.130 (0.068)	0.134 (0.077)
Bladder wall	0.018 (0.013)	0.018 (0.010)
Spleen	0.034 (0.023)	0.034 (0.019)
Bone marrow (image-based)	0.011 (0.010)	0.011 (0.020)
Bone marrow (blood-based)	0.031 (0.010)	0.032 (0.010)
Brain	0.002 (0.001)	0.003 (0.004)
Heart	0.013 (0.004)	0.014 (0.003)
Intestine	0.002 (0.001)	0.002 (0.001)
Adrenals	0.007 (0.002)	0.010 (0.018)
Liver	0.034 (0.021)	0.035 (0.018)
Gall bladder wall	0.004 (0.002)	0.004 (0.002)
Prostate	0.003 (0.002)	0.003 (0.002)
Lungs	0.058 (0.072)	0.049 (0.055)
Stomach wall	0.005 (0.005)	0.004 (0.003)
Pancreas	0.002 (0.001)	0.002 (0.001)
Ureters	0.002 (0.001)	0.002 (0.001)

Data were available for 13 patients at Cycle 1, 11 patients at Cycle 2 and 10 patients at Cycle 3

^a^Due to hydronephrosis of the right kidney of one patient, the absorbed dose to the left kidney only was used for this patient

The cumulative image-based absorbed doses in key at-risk organs were evaluated across all cycles for each individual patient (Supplemental Table S2). In Cohort A, these ranged across the three patients from 5.86 to 8.20 Gy for the kidney, 1.25–5.53 Gy for the salivary glands, and 0.14–0.87 Gy for the bone marrow. In Cohort B, the cumulative absorbed doses ranged across the 10 patients from 1.52 to 8.33 Gy for the kidney, 0.79–3.40 Gy for the salivary glands, and 0.03–0.42 Gy for the bone marrow. All cumulative absorbed doses in all patients in both dosing cohorts were below the historical external beam radiation therapy (EBRT) organ dose limits [30, 31, 32, 33]. Based on the cumulative absorbed doses, the maximum administered activity that would have been required for each patient to reach the EBRT kidney dose limit of 23 Gy was extrapolated. This ranged from 45 to 128 GBq across the 13 patients, equivalent to 6–17 treatments cycles at 7.4 GBq (Supplemental Figure S2).

### Dosimetry in tumours

For the Cycle 1 tumour dosimetry, 36 lesions (27 bone and 9 lymph nodes) were analysed using the activity-based method, while 51 lesions (39 bone and 12 lymph nodes) were evaluated using the anatomy-based method. Eighteen lesions were common to both methods; for these common lesions, the activity outlining method resulted in lesion volumes 60% smaller than those obtained using the anatomy-based method. For each method, the mean tumour dose was determined by averaging the maximum tumour dose across all patients.

When using the activity method, the mean (SD) maximum tumour absorbed dose in Cycle 1 was 8.87 (6.71) Gy/GBq (range across patients, 2.00–25.7 Gy/GBq). When using the anatomy method, smaller maximum tumour absorbed doses were observed, with a mean (SD) estimate of 2.38 (1.78) Gy/GBq (range, 0.37–6.67 Gy/GBq)(Supplemental Figure S3).

**Fig. 3:**
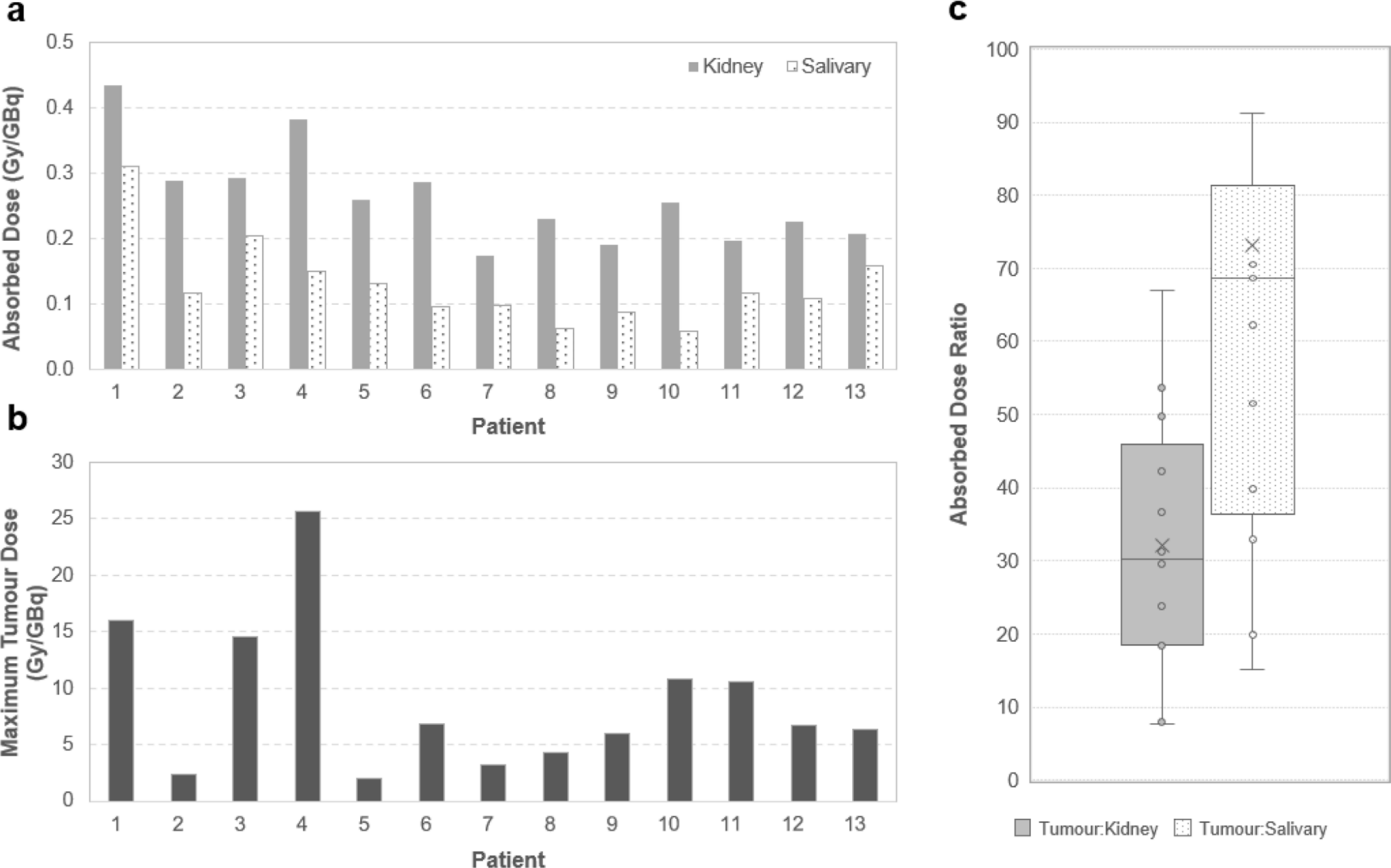
Absorbed Doses of ^177^Lu-rhPSMA-10.1 in Kidneys, Salivary Glands, and Tumours (Cycle 1). (**a**) patient level kidney and salivary gland absorbed dose, (**b**) maximum tumour dose per patient using activity-based method, and (**c**) boxplots of resulting tumour-to-kidney and tumour-to-salivary gland absorbed dose ratios

As detailed in Fig. 3, when using the activity method data, the mean therapeutic index presenting the ratio of tumour-to-kidney absorbed doses was 32.1 (range, 7.7–67), while the mean tumour-to-salivary gland ratio was 73.2 (range, 15–187).

**Fig. 4:**
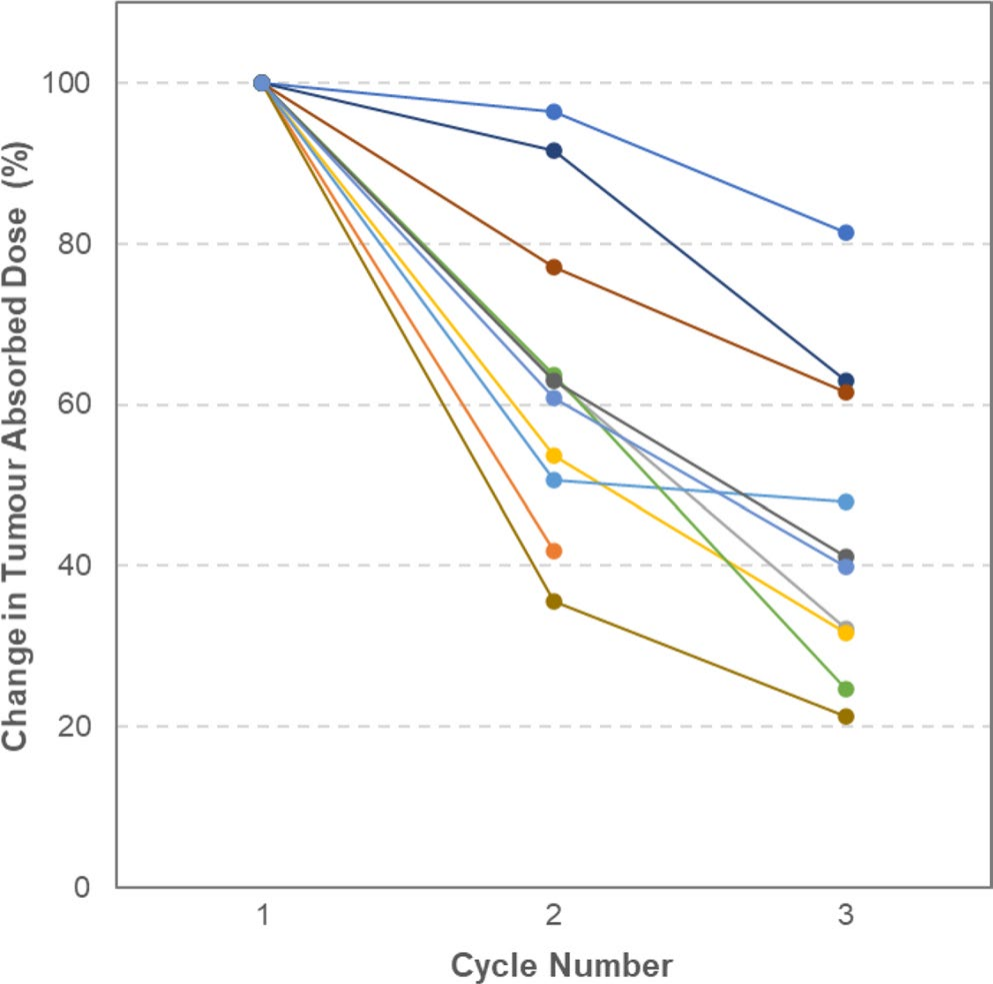
Tumour Absorbed Dose Change with Cycle. Mean percentage change in tumour absorbed dose relative to Cycle 1 for each patient who received at least two treatment cycles

Tumour absorbed doses decreased with each cycle in every one of the 11 patients where multiple cycles of tumour dosimetry data were available. Figure 4 presents the mean percentage change in anatomy-based tumour dose relative to Cycle 1 for each patient. Absorbed doses in Cycle 2 were on average 37% lower than Cycle 1 values, while Cycle 3 absorbed doses were on average 56% lower than Cycle 1 values.

### Effective half-life

**Fig. 5:**
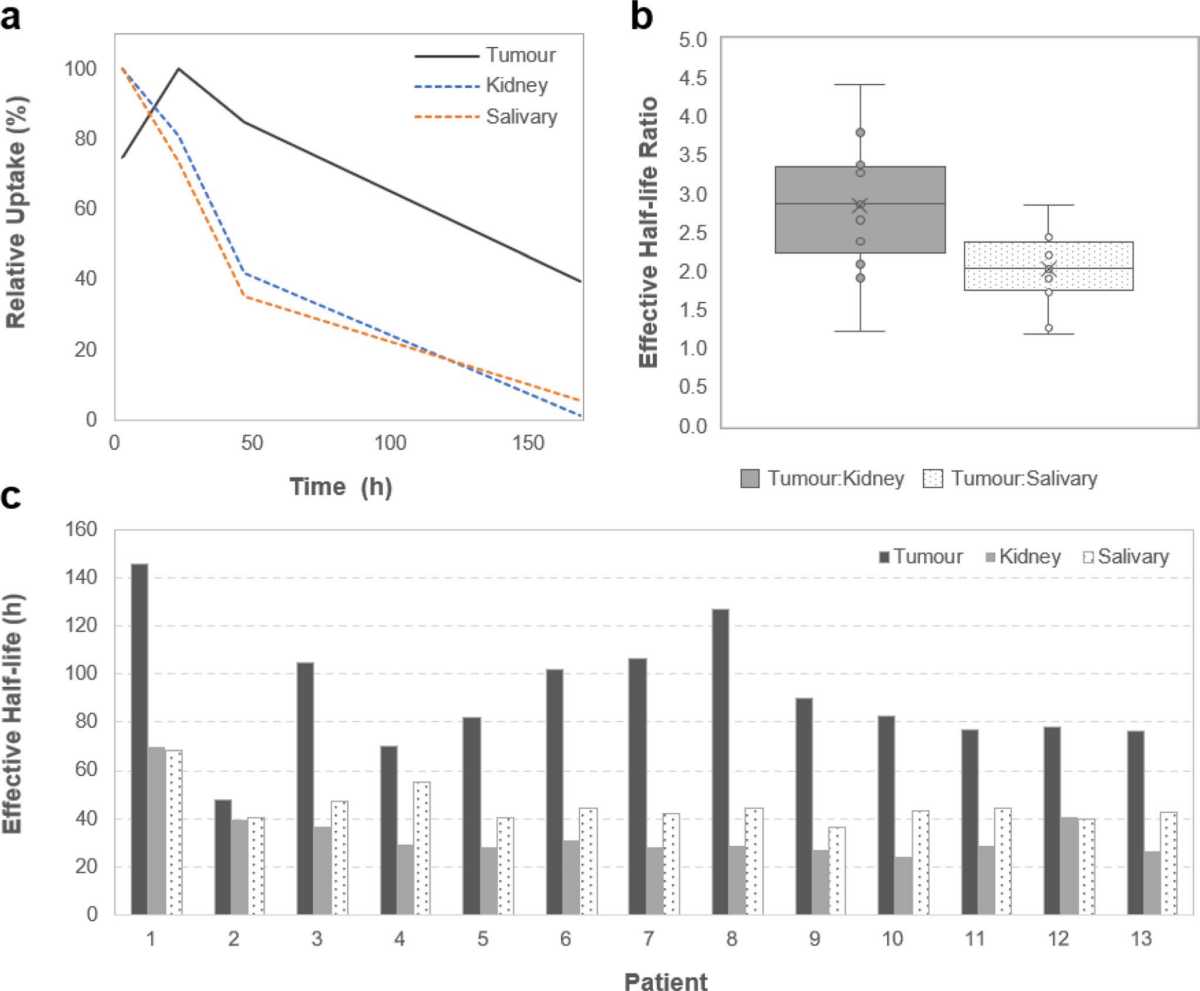
Image-based Pharmacokinetics of ^177^Lu-rhPSMA-10.1 in Tumours, Kidneys, and Salivary Glands. (**a**) presents the uptake relative to peak for ^177^Lu-rhPSMA-10.1 in tumours, kidneys and salivary glands over the course of the study, (**b**) presents the effective half-life of ^177^Lu-rhPSMA-10.1 in tumours, kidneys and salivary glands across all cycles in each patient, and (**c**) shows the ratio of these tumour-to-normal organ half-lives

Based on exponential fits to image-derived time–activity washout data in individual tissues (Fig. 5), the biological and effective half-lives of ^177^Lu-rhPSMA-10.1 in tumours and normal organs were estimated using the mean data from all patients across all cycles (Supplemental Table S3). The mean biological half-life of ^177^Lu-rhPSMA-10.1 was 338 h in tumours, 44.9 h in kidneys and 64.6 h in salivary glands resulting in effective half-lives of 91.4, 33.7 and 45.4 h, respectively.

### Blood-based pharmacokinetics

As shown in Supplemental Figure S4, the radioactivity concentration of ^177^Lu-rhPSMA-10.1 in venous whole blood samples from each patient over the whole evaluation period indicate that ^177^Lu-rhPSMA-10.1 is rapidly cleared within the first two hours post-injection. The effective half-life in blood, based on mean patient levels, was 2.2 (0.5) hours.

## Discussion

Here, we evaluated radiation dosimetry of ^177^Lu-rhPSMA-10.1, a novel radiohybrid PSMA-targeted RLT, in a population of patients with heavily pre-treated mCRPC enrolled in the BET-PSMA-121 study.

Our data show a substantially higher radiation dose of ^177^Lu-rhPSMA-10.1 is delivered to tumours (mean, 8.87 Gy/GBq) than to at-risk normal organs such as the kidneys (0.27 Gy/GBq) and salivary glands (0.13 Gy/GBq). A key factor contributing to the increased absorbed dose in tumours is its relatively high retention time (as represented by the effective half-life). Consistent with preclinical data that show the longer retention of ^177^Lu-rhPSMA-10.1 in tumours compared with normal organs [21], our data show the mean effective half-life of ^177^Lu-rhPSMA-10.1 in tumours is approximately double that in salivary glands and nearly three times as long as in the kidneys. ^177^Lu-rhPSMA-10.1 was selected for clinical development due to its balance of several factors, including an optimised degree of human serum albumin binding, charge, lipophilicity, and other properties impacting uptake, internalisation, and long-term retention. This optimisation is believed to promote its efficient clearance from healthy organs while preserving high levels of accumulation in tumours [19]. Compared with reported data for the tumour effective half-life of ^177^Lu-PSMA-617 (61 h) and ^177^Lu-PSMA-I&T (43 h), our estimate for ^177^Lu-rhPSMA-10.1 (91 h) suggests ^177^Lu-rhPSMA-10.1 offers notably longer retention in tumours than achieved with other ^177^Lu-labelled PSMA RLT agents [34].

Confirming previous preclinical and pretherapeutic dosimetry data that show a favourable tumour-to-kidney therapeutic index for ^177^Lu-rhPSMA-10.1 [21, 22], we show here that up to 67-fold greater dose of ^177^Lu-rhPSMA-10.1 is delivered to the tumour relative to the kidney. Moreover, during Cycle 1, the absorbed dose of ^177^Lu-rhPSMA-10.1 in the kidneys was 0.27 Gy/GBq. This compares favourably with data reported for the approved ^177^Lu-labelled PSMA RLT, ^177^Lu-PSMA-617; Data from a dosimetry substudy of the Phase III VISION trial show the absorbed dose of ^177^Lu-PSMA-617 in the kidneys to be 0.43 Gy/GBq [35]. As discussed, it is likely that higher tumour-to-kidney absorbed dose ratios, as demonstrated here for ^177^Lu-rhPSMA-10.1, will facilitate higher tumour radiation doses to be achieved relative to the safe kidney limits. These limits are likely to be established specifically for different patient populations based on risk factors and remaining life expectancy.

Salivary glands are also considered a significant at-risk organ during ^177^Lu-labelled PSMA RLT, with both PSMA-specific and nonspecific mechanisms thought to contribute to the effect [5, 36]. Again, the present data demonstrate the efficient clearance of ^177^Lu-rhPSMA-10.1 from normal organs relative to tumours [22], with a tumour-to-salivary gland ratio of up to 187. We show the absorbed dose of ^177^Lu-rhPSMA-10.1 in the salivary glands to be 0.13 Gy/GBq, which compares favourably with data reported from the VISION substudy for ^177^Lu-PSMA-617 (0.63 Gy/GBq) [35].

Two methods (image-based and blood-based) were employed here to estimate absorbed dose to the bone marrow, a further key at-risk organ in ^177^Lu-labelled PSMA RLT given the potential for haematological toxicity. Notwithstanding the practical challenges associated with each method, both have their own advantages and limitations when it comes to reported accuracy [37, 38], and while the superiority of one method over another is yet to be determined, understanding the association between absorbed dose and haematologic toxicity will be critical to this. In the present study, the blood-based method gave on average a 3-fold higher estimation of bone marrow dose than the image-based method (0.032 vs. 0.011 Gy/GBq). The blood-based absorbed dose compared equivalently with data reported from the VISION dosimetry substudy for ^177^Lu-PSMA-617 (0.035 Gy/GBq) [35].

While there are limited prospective data directly linking dosimetry results with patient outcomes, dosimetry remains essential in the development and optimisation of radiopharmaceuticals. It facilitates the estimation of expected radiation doses to tumours relative to normal organs which furthers the understanding and assessment of a radiopharmaceutical’s safety profile and potential efficacy. However, dosimetry is not without challenges. As shown in a recent meta-analysis of dosimetry of ^177^Lu-labelled PSMA RLT, significant variation in tumour doses is observed across studies [28]. As inter-study variations in the methodology likely contribute to this disparity, we evaluated the absorbed tumour dose of ^177^Lu-rhPSMA-10.1 during the first treatment cycle using two distinct methods to select and contour tumour lesions. To date, most dosimetry analyses have focused on measuring absorbed dose in tumours that have been specifically selected for their high target expression, and often utilise a thresholding approach to outline tumours rather than CT. In the present study we evaluated tumour uptake in two different populations of lesions. We performed analyses using an activity-based methodology consistent with the literature in which lesions were specifically selected for their higher uptake using the 168-hour SPECT/CT, and contoured via a 41% SUV_max_ threshold on PSMA PET/CT, then manually transferred onto SPECT/CT. We also opted for an unbiased random selection of lesions, using RECIST baseline criteria as the basis for selection, which were manually contoured using CT. Our data show the substantial impact that these two approaches for tumour selection and contouring have on reported absorbed dose estimates; when using the activity method, the mean maximum tumour was approximately 3.7 times higher than when using the anatomy method (8.87 vs. 2.38 Gy/GBq, respectively). Relative volume differences between the two methods were also evident, with PET thresholding resulting in tumour volumes that were, on average, 60% smaller than those obtained using CT morphology across the same population of lesions. This disparity likely reflects the influence of PSMA-avid subregions of the tumour on the boundaries delineated by PET thresholding. This highlights an important limitation of comparing tumour absorbed doses across studies that employ varying methods to select and contour tumours for analysis. The field should therefore work towards establishing reference standards for this aspect of tumour dosimetry.

A further compelling reason for comprehensive dosimetry analyses is to support the refinement of dosing strategies and inform the overall clinical development programme. The present Phase I data indicate that the cumulative absorbed dose to the kidney did not reach beyond 36% of the EBRT dose limit for the kidney (23 Gy) [30], and although we evaluated only a maximum of three cycles here, the potential exists for high cumulative activities to be administered in Phase II in order to deliver higher doses to the tumour with the goal of maximising efficacy. An additional important observation from the present study is the decrease in absorbed dose in tumours in the second and then again in the third treatment cycle, likely due to the reduction in PSMA expression correlating with dosimetric effect. However, other factors including changes to the tumour vasculature and physical volume may also contribute to this effect. Given this observation, it is proposed that the optimal dosing schedule for a patient undergoing RLT with agents such as ^177^Lu-rhPSMA-10.1 is unlikely to be a fixed dose delivered at a fixed interval. The data here suggest that administering a higher proportion of the activity in early cycles, when the ratio between tumour and normal organs is at its highest, will result in a higher cumulative absorbed dose to tumours without altering the absorbed dose to normal organs. The impact of this approach on efficacy will be evaluated in the ongoing Phase II component of this study.

In addition to the challenges of dosimetry outlined above, several limitations to the present work exist. Firstly, the MIRD methodology employed in the study assumes a uniform activity distribution and therefore does not consider any absorbed dose heterogeneity within organs. Additionally, while individual patient organ masses were scaled to reflect deviations from standard phantom sizes, other critical aspects of patient-specific anatomy, such as organ shapes, positions, and inter-organ distances, were not incorporated into the dosimetry calculations. Typical of studies of this type and phase, our analyses were conducted in only a small number of patients, and among only a selection of tumours rather than across the whole tumour burden which may mean that the heterogeneity of PSMA expression expected in a real-world population of patients with prostate cancer [7, 8, 9] may not be accurately represented. However, the results of the present analysis have informed the dosing schedule for the ongoing Phase II component of this study which is evaluating the safety and efficacy of ^177^Lu-rhPSMA-10.1 in a larger number of patients and at significantly higher cumulative activities.

## Conclusion

In summary, this prospective Phase I study in patients with mCRPC demonstrates that ^177^Lu-rhPSMA-10.1 has a high therapeutic index as defined by the ratio of tumour-to-kidney and tumour-to-salivary gland absorbed doses. The effective half-life suggests a prolonged retention in tumours which likely drives the high tumour absorbed dose. Furthermore, low cumulative absorbed doses to at-risk organs, plus the observation of a decreasing tumour absorbed dose with each subsequent treatment cycle shows potential for a front-loaded dosing schedule alongside a high cumulative administered activity in subsequent studies of ^177^Lu-rhPSMA-10.1.

## Electronic supplementary material

Below is the link to the electronic supplementary material.

Supplementary Material 1

## Data Availability

The datasets generated during and/or analysed during the current study are available from the corresponding author on reasonable request.
