# Supplementary material for: Organ and tumour dosimetry of 177Lu-rhPSMA-10.1, a novel PSMA-targeted therapy: results from a Phase I trial
Source: Eur J Nucl Med Mol Imaging. 2025 May 6;52(12):4414–24. doi: 10.1007/s00259-025-07313-z (PMC12491383; doi:10.1007/s00259-025-07313-z)
Supplement: Supplementary file 1 — Supplementary Material 1 [file 259_2025_7313_MOESM1_ESM.docx]

**SUPPLEMENTAL MATERIALS**

**Dosimetry Methodology**

All patients underwent multi-bed single-photon emission computed tomography (SPECT)/CT at 3-, 24-, 48- and 168-hours post-^177^Lu-rhPSMA-10.1 administration during each treatment cycle. The acquisition parameters are shown in Supplemental Table S1. All images were centrally reconstructed in 3D-RD-S using the iterative Ordered Subsets Expectation-Maximization (OSEM) algorithm with 40 iterations and 5 subsets. Attenuation, scatter, and the Collimator-Detector Response Function (CDRF) were modelled during the iterative process using a rotation-based projector. CT-based attenuation compensation was performed using the attenuation map generated from the CT image. Scatter was modelled using the Effective Source Scatter Estimation (ESSE) method.

Reconstructed images were converted to activity concentration units (Becquerel (Bq)/millilitre (mL)) using the calibration factor generated for each individual imaging system. Organ masses were individually determined using CT-based delineations and longitudinal SPECT activity statistics were based on these same delineations.

Time integrated activity coefficients (TIACs) were calculated for each target tissue. This was done by fitting each time–activity curve with an exponential function (mono, bi, or exponential uptake and washout) and integrating from time 0 to infinity. The simpler function was preferred provided the fit was adequate (R^2^<0.9).

The TIAC in the bone marrow was derived from contours drawn around the marrow inside lumbar vertebrae L3–L5. Where lesions were present in the lumbar area, contours were drawn around either the femur heads, scapula or sternum depending on the anatomical coverage. The TIAC was then divided by the marrow mass in this region to give the TIAC concentration. The total bone marrow TIAC was be obtained as the product of this concentration and the standard International Commission on Radiological Protection (ICRP) bone marrow mass.

The S-value methodology was used to estimate the target tissue dose. The S-values were calculated from specific absorbed fraction (SAF) values that describe the fraction of energy of a given particle type (e.g., electron, photon, or alpha particle) at a given energy emitted uniformly from a source tissue that is deposited in a target tissue. The SAF values were obtained from the ICRP and were originally calculated using Monte Carlo Simulation based upon standard or reference geometries for male phantoms. The S-values were calculated from these SAF values and radionuclide decay data tabulated in ICRP-133 and ICRP-107 [1,2], respectively. Since the lacrimal glands are not one of the ICRP pre-defined target tissues, the dose to the lacrimal glands was calculated as the self-dose to a 1.2 cm diameter sphere (~0.93 g with a density of 1.03 g/cm^3^).

**Supplemental Table S1** Imaging Parameters for Dosimetry

| Image Parameter | Setting |
| --- | --- |
| Number of projections | 120 |
| Orbit type | Body contour |
| Projections per head | 60 |
| Number of angles | 60 |
| Angular step | 3^◦^ |
| Matrix | 128 × 128 |
| Time per step | 15 seconds |
| Mode | Step and shoot |
| Collimator | Medium energy |
| Energy windows (peak) | 20% at 113 keV and 208 keV |
| Energy windows for scatter correction | 166.4–187.2 and 228.8–249.6 keV |
| Reconstruction | OSEM (3D-RD-S), 40i5s |

**Supplemental Table S2** Cumulative Absorbed Doses for At-risk Organs

|  | | | | **Cumulative Absorbed Dose,**  **Gy (% of dose limit*)** | | |
| --- | --- | --- | --- | --- | --- | --- |
|  | **Patient** | **Cycles received** | **Cumulative administered activity, GBq** | **Kidney** | **Salivary Glands** | **Bone Marrow** |
| **Cohort A** | 1 | 3 | 16.20 | 8.20 (36) | 5.53 (23) | 0.14 (7.0) |
|  | 2 | 2 | 11.57 | 5.86 (25) | 1.25 (5.2) | 0.14 (7.0) |
|  | 3 | 3 | 16.58 | 6.15 (27) | 2.96 (12) | 0.87 (44) |
| **Cohort B** | 4 | 3 | 22.94 | 8.33 (36) | 2.87 (12) | 0.13 (6.5) |
|  | 5 | 3 | 22.29 | 6.25 (27) | 2.85 (12) | 0.07 (3.5) |
|  | 6 | 3 | 22.59 | 7.31 (32) | 2.87 (12) | 0.42 (21) |
|  | 7 | 3 | 22.29 | 4.02 (17) | 2.88 (12) | 0.05 (2.5) |
|  | 8 | 3 | 22.86 | 5.18 (23) | 1.17 (4.9) | 0.24 (12) |
|  | 9 | 3 | 22.10 | 5.18 (23) | 2.00 (8.3) | 0.05 (2.5) |
|  | 10 | 3 | 23.30 | 4.29 (19) | 0.82 (3.4) | 0.03 (1.5) |
|  | 11 | 1 | 7.80 | 1.52 (6.6) | 0.88 (3.7) | 0.23 (11) |
|  | 12 | 1 | 7.46 | 1.71 (7.4) | 0.79 (3.3) | 0.04 (2.0) |
|  | 13 | 3 | 22.23 | 4.63 (20) | 3.40 (14) | 0.05 (2.5) |
| *Estimates based on historical external beam radiation therapy limits of 23 Gy for the kidney [3], 24 Gy for salivary glands [4], and 2 Gy for bone marrow [5,6]. | | | | | | |

**Supplemental Table S3** Biological and Effective Half-lives in Tumours, Kidneys, and Salivary Glands

| **Patient** | **Effective Half-life, hours** | | | **Biological Half-life, hours** | | |
| --- | --- | --- | --- | --- | --- | --- |
|  | **Tumours^a^** | **Kidneys** | **Salivary Glands** | **Tumours^a^** | **Kidneys** | **Salivary Glands** |
| 1 | 145.8 | 69.6 | 68.6 | 1692.5 | 123.6 | 120.2 |
| 2 | 48.1 | 39.4 | 40.5 | 68.8 | 52.3 | 54.2 |
| 3 | 104.8 | 36.6 | 47.4 | 305.1 | 47.5 | 67.5 |
| 4 | 70.1 | 29.3 | 55.3 | 124.9 | 35.9 | 84.7 |
| 5 | 82.1 | 28.5 | 40.3 | 168.9 | 34.6 | 54.0 |
| 6 | 102.0 | 31.2 | 44.3 | 282.5 | 38.7 | 61.3 |
| 7 | 106.1 | 27.9 | 42.1 | 316.6 | 33.9 | 57.1 |
| 8 | 126.6 | 28.6 | 44.4 | 611.3 | 34.9 | 61.5 |
| 9 | 89.7 | 26.8 | 36.7 | 204.8 | 32.3 | 47.7 |
| 10 | 82.4 | 24.3 | 43.1 | 170.2 | 28.7 | 59.0 |
| 11 | 76.8 | 28.9 | 44.5 | 148.2 | 35.3 | 61.7 |
| 12 | 78.0 | 40.6 | 40.1 | 152.4 | 54.5 | 53.6 |
| 13 | 76.2 | 26.4 | 42.4 | 145.7 | 31.6 | 57.8 |
| **Mean** | **91.4** | **33.7** | **45.4** | **337.8** | **44.9** | **64.6** |
| **SD** | **25.6** | **11.9** | **8.3** | **429.5** | **25.0** | **18.9** |

^a^Tumour half-life values were derived from the anatomy-based method and averaged across all tumours and cycles (n = 137).

**Supplemental Fig S1** Representative PET/CT and SPECT/CT images illustrating the transfer of the tumour contour from PET to SPECT and the subsequent margin adjustments for the activity-based method


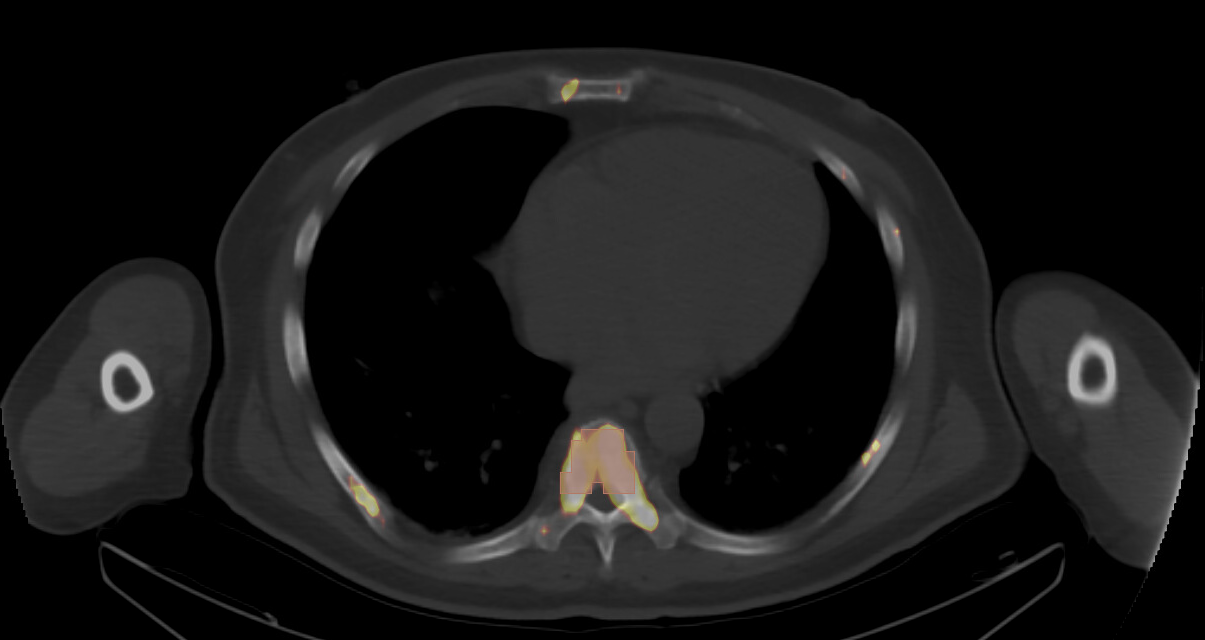

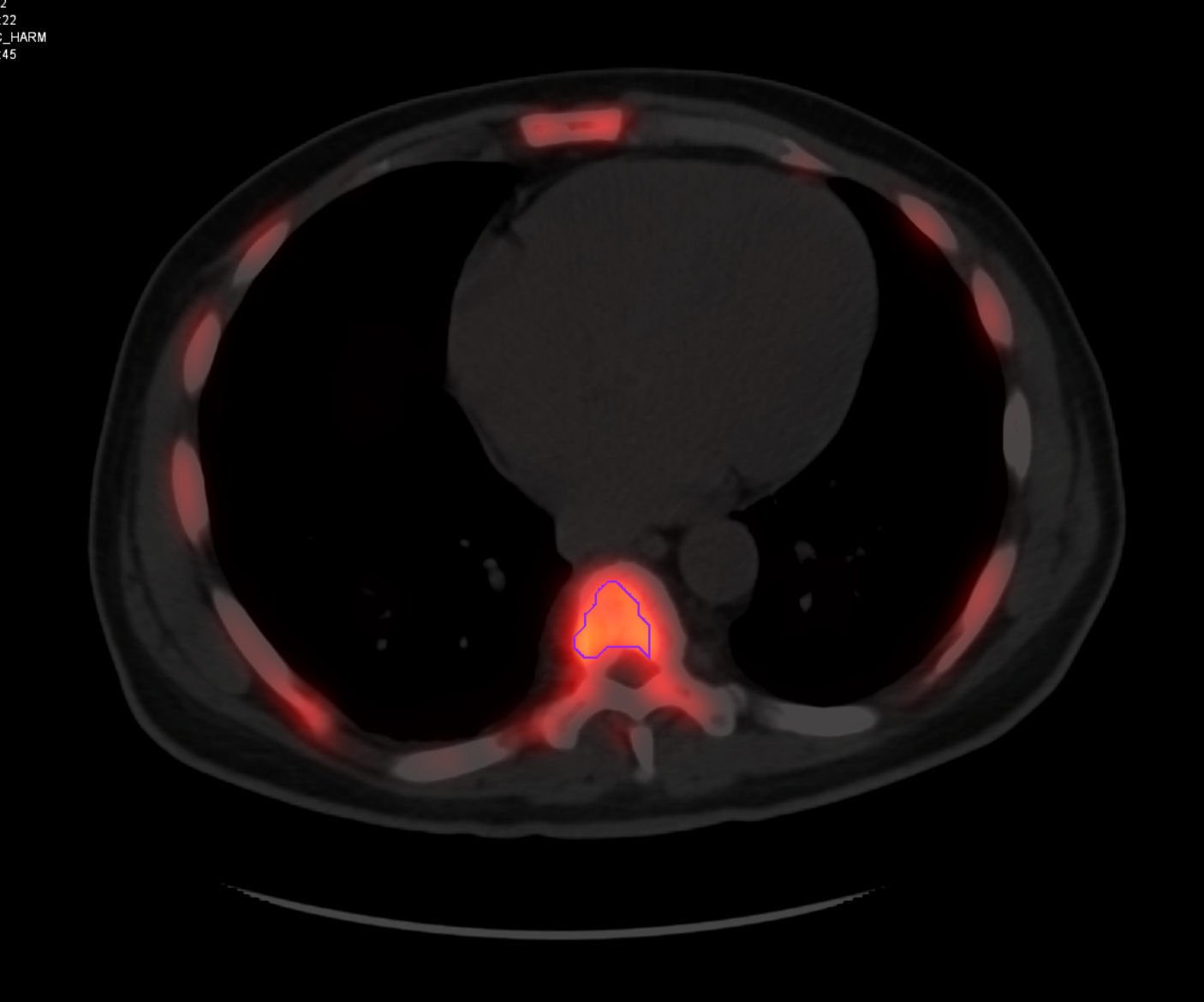


**PET/CT Fused Axial**

**SPECT/CT Fused Axial**

**Supplemental Fig S2** Projected Maximum Administered Activity Needed to Reach a 23 Gy Kidney Absorbed Dose Limit

**Supplemental Fig S3** Maximum Tumour Dose per Patient Using the Activity *vs* Anatomy Method

**Supplemental Fig S4** ^177^Lu-rhPSMA-10.1 Kinetics in Blood Samples (a) and Effective Half-life in Blood Based on Mean Patient-level Values (b)​


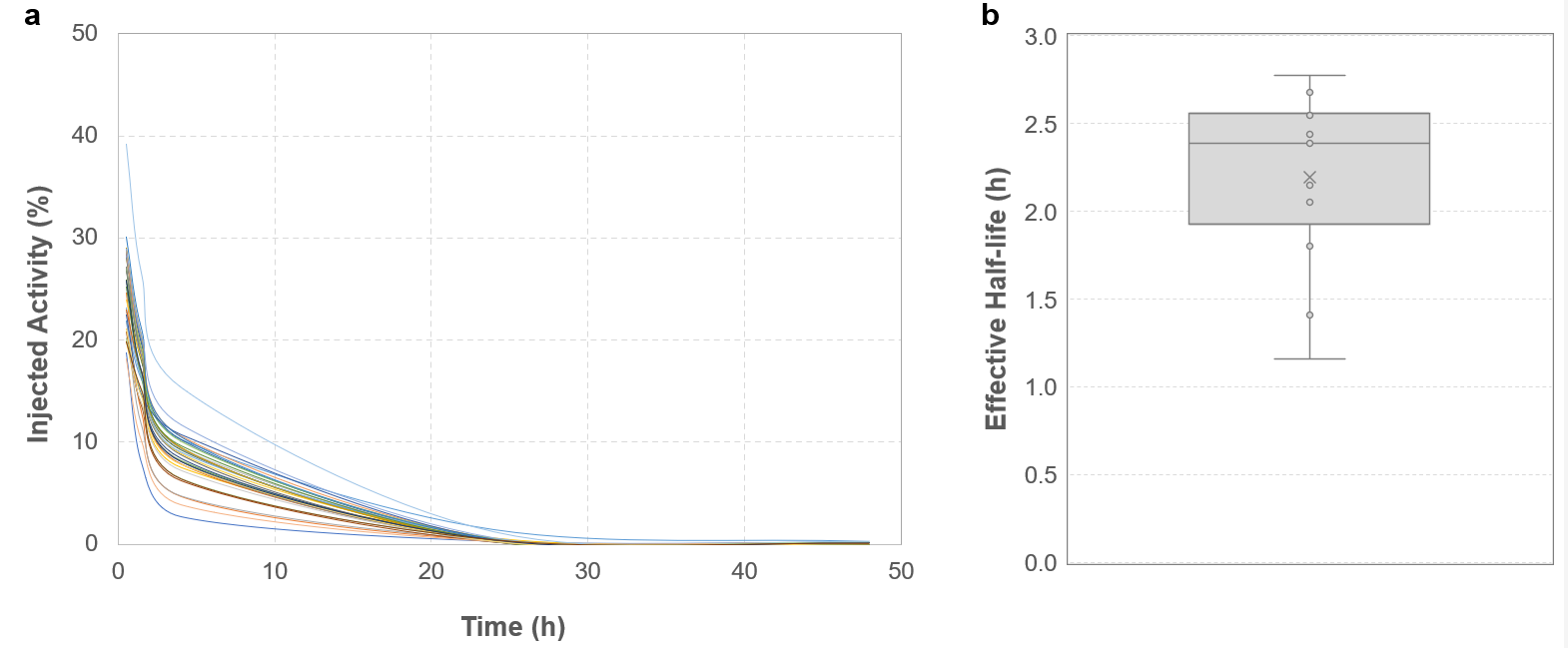


Each line in plot (a) represents an individual patient cycle.

**SUPPLEMENTAL REFERENCES**

1. ICRP. The ICRP computational framework for internal dose assessment for reference adults: specific absorbed fractions. ICRP Publication 133. Ann ICRP 2016;45:1–74.

2. ICRP. Nuclear decay data for dosimetric calculations. ICRP Publication 107. . Ann ICRP. 2008;38.

3. Dawson LA, Kavanagh BD, Paulino AC, Das SK, Miften M, Li XA, et al. Radiation-associated kidney injury. Int J Radiat Oncol Biol Phys. 2010;76:S108-15. doi:10.1016/j.ijrobp.2009.02.089.

4. Eisbruch A, Ten Haken RK, Kim HM, Marsh LH, Ship JA. Dose, volume, and function relationships in parotid salivary glands following conformal and intensity-modulated irradiation of head and neck cancer. Int J Radiat Oncol Biol Phys. 1999;45:577-87. doi:10.1016/s0360-3016(99)00247-3.

5. DeNardo GL, Schlom J, Buchsbaum DJ, Meredith RF, O'Donoghue JA, Sgouros G, et al. Rationales, evidence, and design considerations for fractionated radioimmunotherapy. Cancer. 2002;94:1332-48. doi:10.1002/cncr.10304.

6. International Commission on Radiological Protection (ICRP). ICRP statement on tissue reactions / early and late effects of radiation in normal tissues and organs – threshold doses for tissue reactions in a radiation protection context. ICRP Publication 118. Ann ICRP 2012;41.
